# Post-esophagectomy Anastomosis Leak: A 10-Year Experience at a Specialized Center for Cancer Surgeries in Pakistan

**DOI:** 10.7759/cureus.34777

**Published:** 2023-02-08

**Authors:** Ahsan Shafiq, Junaid Azad, Shan-e-Zahra Batool, Usman Ismat Butt, Muhammad Umar, Aamir Syed, Shahid Khattak

**Affiliations:** 1 Surgical Oncology, Shaukat Khanum Memorial Cancer Hospital and Research Centre, Lahore, PAK; 2 Surgery, Services Hospital Lahore, Lahore, PAK

**Keywords:** oesophageal cancer, specialized-services, ec- esophageal cancer, transthoracic esophagectomy, the: transhiatal esophagectomy

## Abstract

Esophageal cancer has been reported to be the seventh most common cancer and the sixth most common cause of mortality. Use of advanced diagnostic techniques has increased the detection of preoperative metastases and resulted in better patient selection for further management by curative surgery. We carried out a study to evaluate the outcome of esophagectomy at our institute in terms of acute leak, mortality and hospital stay. We also looked at various preoperative, intraoperative and postoperative risk factors contributing to leak after esophagectomy.

We evaluated 589 patients during the period from January 2009 to December 2019. All these patients underwent elective esophagectomy for esophageal cancer at our hospital. Out of these, leak was seen in 30 patients (5.1%). We found no statistically significant difference when evaluating patient and tumour characteristics of patients who developed leak against those who did not. We also didn’t find any significant difference in intraoperative or postoperative factors between the two groups.

Proper preoperative evaluation and optimization are necessary to overcome various patient co-morbidities. On the basis of our study we conclude that when performed in high-volume centers with an adequately trained multi-disciplinary team approach, esophagectomy for carcinoma has a good outcome.

## Introduction

Esophageal cancer has been reported to be the seventh most common cancer. It is reported to be the sixth most common cause of mortality [1]. Risk factors for esophageal carcinoma include gastroesophageal reflux disease (GERD), smoking, achalasia, obesity, infection by virus, poverty, and genetic factors [2].

Use of advanced diagnostic techniques has increased the detection of preoperative metastases and therefore resulted in better patient selection for further management by curative management. Other developments in endoscopic field have also advanced not only diagnostic but therapeutic capabilities as well. Advances in surgical technique and intensive care have resulted in improvement in survival in resectable cases [3]. The reported morbidity of esophagectomy despite improvements is still reported to be as high as 30%. However five-year survival of these patients is only 20% [4].

The main surgical treatment option for resectable esophageal cancer is esophagectomy. It is a complex undertaking having considerable morbidity and mortality. The incidence of esophageal cancer is increasing worldwide. Despite multidisciplinary care and improvements in treatment modalities, the outcome remains poor. Neoadjuvant chemoradiation prior to surgical resection is the current approach. It has resulted in improved survival in patients with early disease. Different approaches are used to perform this procedure. Trans-hiatal, Ivor Lewis and McKeown are types of primary open surgery. Minimal access laparoscopy and thoracoscopic approach are also used while lymphadenectomy may be one-, two-, or three-field depending upon the nature of the disease. Because of the invasiveness of the procedure it is associated with many complications which result in reduction of survival [5].

In this study we have evaluated the outcome of esophagectomy at our institute in terms of acute leak, mortality and hospital stay which were carried out over a 10-year period from January 2009 to December 2019. We also looked at various preoperative, intraoperative and postoperative risk factors contributing to leak after esophagectomy.

## Materials and methods

We included patients who underwent esophagectomy at Shaukat Khanum Memorial Cancer Hospital (IRB approval ex-11-01-23-01). Cases carried out between January 2009 and December 2019 were reviewed.

American Joint Committee on Cancer (AJCC) 8th edition classification was used to stage the patients. A multi-disciplinary team meeting was done to discuss management of all patients. Neoadjuvant chemo-radiotherapy was done for patients having locally advanced disease (patients with T3 or above and/or N1 disease). External beam radiation between 45 and 55Gy with cisplatin and 5FU for given to patients as part of neoadjuvant therapy. Four weeks after the completion of the neoadjuvant, restaging scan was done. Patients found to have resectable disease then underwent elective esophagectomy. Intraoperative and postoperative records were maintained on a computerized data system. All relevant data were recorded and 90-day mortality was noted.

For gastroesophageal junction tumours, esophagectomy was performed via a transhiatal approach while for mid/lower esophageal tumours a transthoracic approach (three-stage or Ivor Lewis esophagectomy) approach was used. During surgery, the tumour and its adjacent lymph nodes were removed en bloc. We noted the age of the patient, their gender, endoscopic level of tumor, differentiation of tumor, type of tumor, tumor T and N stage and operative approach used. We made use of SPSS® version 22.0 for Windows™ (IBM Corp., Armonk, NY, USA) for data analysis. Frequencies and percentages were used for description of categorical variables. Quantitative variables were described as median with range. To compare categorical data chi-square test was used for binominal variables and one-way analysis of variance for tri and tetra nominal variables. A value of p less than 0.05 was taken as significant.

## Results

We evaluated patients undergoing esophagectomy during the period from January 2009 to December 2019. All these patients underwent elective esophagectomy for esophageal cancer at our hospital. Out of these, leak was seen in 30 (5.1%). We found no statistically significant difference when evaluating patient and tumour characteristics of patients who developed leak or not. We also didn’t find any significant difference in intraoperative or postoperative factors between the two groups (Table 1).

**Table 1:** Baseline patient and tumour characteristics

Variable	Leak= yes	Leak = no	P- value
Gender			
Male	18	311	0.640
Female	12	248	
Type of cancer			
Squamous cell carcinoma	24	442	0.944
Adeno carcinoma	5	109	
Other	1	8	
Site of cancer			
Upper	0	11	0.499
Mid	5	109	
Lower	25	439	
Tumour stage			
T1	0	30	0.519
T2	1	459	
T3	26	25	
T4	3	45	
Node stage			
N0	6	176	0.126
N1	18	314	
N2	6	69	
Neoadjuvant therapy			
Yes	30	535	0.247
No	0	24	
Type of surgery			
3 stage	23	360	0.254
2 stage	1	173	
Trans Hiatal	6	26	
Pre-operative Haemoglobin			
Less than 10 g / dl	1	49	0.299
More than 10 g / dl	29	510	
Intra-operative pressor			
Yes	8	101	0.232
No	22	458	
intra-operative Bleeding			
Less than 250 ml	29	478	0.086
More than 250 ml	1	81	
90 day mortality			
Yes	4	10	0.0038
No	26	549	

The average age of patients undergoing surgery at our hospital was 49.53 years (SD±11.029 years). A little more than half (55.9%) of the patients were male. Tumours of the lower esophagus were the most commonly seen (77.9%). The most common type was however squamous cell carcinoma (79.1%). The most commonly performed surgery at our set up was three-stage esophagectomy (65%). The average hospital stay was 13.82±5.45 days. Our 90-day mortality was 2.4%.

Of the 30 patients who developed postoperative leak all were explored. Almost half the patients underwent re-anastomosis (Table 2). There were four mortalities in the patients who developed leak.

**Table 2:** Breakdown of post leak intervention and mortality by type of procedure done

Intervention	Number of cases	Mortalities
Exploration and re-anastomosis	14	3
Exploration and drainage	16	1
Total	30	4

## Discussion

In this retrospective study of patients undergoing esophagectomy at our institute from January 2009 to December 2019, 30 patients developed postoperative leak (5.1%). These patients were analyzed and compared to evaluate the potential risk factors. Incidence of leakage reported in our study is similar to that reported in international literature. Famiglietti et al. in their article published in 2020 have quoted rates of anastomotic leak ranging from 2-17% [6].

The purpose of our study was to evaluate the data of our patients who had undergone surgery for esophageal carcinoma to evaluate postoperative mortality and evaluate various factors influencing the outcome in these patients. We evaluated various patient-, disease- and surgery-related factors influencing the outcomes. We analyzed the probable risk factors of patients developing leak post esophagectomy. However our results failed to show any statistically significant correlation between leak and factors (Table 1).

Acute leak is one of the commonest and most dreaded complications faced after esophagectomy. A number of researchers have worked to identify factors associated with increased risk of leak. A number of factors have been thought to be associated with this risk including patient and surgery related such as history of smoking, adverse pulmonary or cardiac events, three-stage approach, advance tumour stage and nutritional status. It has been documented that leak is associated with worse outcome including increased postoperative mortality, longer need to keep patient nil per oral, and longer hospital and ICU stays [6].

In our study we found that patient-associated or tumour-associated characteristics such as gender, TNM stage, type of tumour or site of tumour had no co-relation with postoperative outcome. Gockel in his risk analysis noted that only site of tumour was significantly related to postoperative morbidity while none of the other preoperative patient or tumor characteristics had any significant correlation [7].

The number of leaks that were seen were more in transthoracic three-stage esophagectomy as compared to transhiatal dissection but this difference was not found to be statistically significant. Schröder in his review found no significant difference in outcome when comparing types of surgeries [8]. Similarly, no significant correlation was seen in patients receiving neoadjuvant therapy as compared to those for whom upfront surgery was performed [9].

When we evaluated for the difference between cases using the blood lost during the procedure (keeping a value of 250ml) we found no statistical difference between the two groups. Boshier et al., in a systematic review and meta-analysis, showed that perioperative blood transfusion was associated with significantly worse long-term survival in esophageal cancer patients undergoing esophagectomy [10]. However our study failed to show similar findings. Similarly, intraoperative pressor use also had no significant effect on the postoperative risk of leak. Walsh et al. in their cohort study showed that intraoperative vasopressor use was not significantly associated with increased odds of postoperative anastomotic leak following open Ivor Lewis esophagectomy [11].

Our results however showed that developing leak was significantly related to 90-day mortality. Fabbi et al. in a review of articles from 1995 to 2019 noted that leakage was associated with significant morbidity and mortality [12].

Our study has a number of limitations. First, our study is a single-center study. Although our center is a specialized center for management of cancer patients, the patients presenting to our center are limited and not representative of the whole population. Second, we evaluated a limited number of variables. Perhaps a more elaborated study might yield some clinically significant relations. 

## Conclusions

On the basis of our study we conclude that when performed in high-volume centers with an adequately trained multi-disciplinary team approach, esophagectomy for carcinoma has a good outcome in terms of postoperative leakage. Proper preoperative evaluation and optimization are necessary to overcome various patient co-morbidities.

## References

[1] Huang J, Koulaouzidis A, Marlicz W (2021). Global burden, risk factors, and trends of esophageal cancer: an analysis of cancer registries from 48 countries. Cancers (Basel).

[2] Yang CS, Chen X, Tu S (2016). Etiology and prevention of esophageal cancer. Gastrointest Tumors.

[3] Harada K, Rogers JE, Iwatsuki M, Yamashita K, Baba H, Ajani JA (2020). Recent advances in treating oesophageal cancer. F1000Res.

[4] Tustumi F, Kimura CM, Takeda FR, Uema RH, Salum RA, Ribeiro-Junior U, Cecconello I (2016). Prognostic factors and survival analysis in esophageal carcinoma. Arq Bras Cir Dig.

[5] Borggreve AS, Kingma BF, Domrachev SA (2018). Surgical treatment of esophageal cancer in the era of multimodality management. Ann N Y Acad Sci.

[6] Famiglietti A, Lazar JF, Henderson H (2020). Management of anastomotic leaks after esophagectomy and gastric pull-up. J Thorac Dis.

[7] Gockel I, Exner C, Junginger T (2005). Morbidity and mortality after esophagectomy for esophageal carcinoma: a risk analysis. World J Surg Oncol.

[8] Takahashi C, Shridhar R, Huston J, Blinn P, Maramara T, Meredith K (2021). Comparative outcomes of transthoracic versus transhiatal esophagectomy. Surgery.

[9] Matsuda S, Kawakubo H, Mayanagi S, Irino T, Kitagawa Y (2021). Surgery and adjuvant therapy after esophagectomy. Ann Esophagus.

[10] Boshier PR, Ziff C, Adam ME, Fehervari M, Markar SR, Hanna GB (2018). Effect of perioperative blood transfusion on the long-term survival of patients undergoing esophagectomy for esophageal cancer: a systematic review and meta-analysis. Dis Esophagus.

[11] Walsh KJ, Zhang H, Tan KS (2021). Use of vasopressors during esophagectomy is not associated with increased risk of anastomotic leak. Dis Esophagus.

[12] Fabbi M, Hagens ER, van Berge Henegouwen MI, Gisbertz SS (2021). Anastomotic leakage after esophagectomy for esophageal cancer: definitions, diagnostics, and treatment. Dis Esophagus.

